# Data-driven survival modeling for breast cancer prognostics: A comparative study with machine learning and traditional survival modeling methods

**DOI:** 10.1371/journal.pone.0318167

**Published:** 2025-04-22

**Authors:** Theophilus Gyedu Baidoo, Hansapani Rodrigo

**Affiliations:** School of Statistical and Mathematical Sciences, The University of Texas Rio Grande Valley, Edinburg, Texas, United States of America; State University of New York at Oswego, UNITED STATES OF AMERICA

## Abstract

**Background** This investigation delves into the potential application of data-driven survival modeling approaches for prognostic assessments of breast cancer survival. The primary objective is to evaluate and compare the ability of machine learning (ML) models and conventional survival analysis techniques, to identify consistent key predictors of breast cancer survival outcomes.

**Methods** This study employs data-driven survival modeling approaches to predict breast cancer survival, including survival-specific methods such as the Cox Proportional Hazards (CPH) model, Random Survival Forests (RSF), and Cox Proportional Deep Neural Networks (DeepSurv), as well as machine learning models like Random Forests (RF), XGBoost, Support Vector Machines (SVM) with an RBF Kernel, and LightGBM. The dataset, sourced from the National Cancer Institute’s Surveillance, Epidemiology, and End Results (SEER) program, comprises 4,024 women diagnosed with infiltrating duct and lobular carcinoma breast cancer between 2006 and 2010. To ensure interpretability across all models, the Shapley Additive Explanation (SHAP) method was applied to RSF, DeepSurv, Random Forests (RF), and XGBoost. This enabled the identification of key predictors influencing breast cancer survival, highlighting consistent factors across models while uncovering unique insights specific to each approach.

**Results** The performance of survival-specific and ML models were evaluated using the Concordance index (C-index), Integrated Brier Score (IBS), mean accuracy, and mean AUC. The CPH model achieved a C-index of 0 . 71 ± 0 . 015 and an IBS of 0 . 08 ± 0 . 006, while RSF demonstrated slightly better discriminatory power with a C-index of 0 . 72 ± 0 . 0117. DeepSurv performed comparably, with a C-index of 0 . 71 ± 0 . 0095 and an IBS of 0 . 09 ± 0 . 0008. Both Cox and RSF models achieved the lowest IBS (0 . 08), indicating accurate survival probability predictions over time. For ML models, RF achieved a mean AUC of 0 . 74 ± 0 . 0021, and XGBoost with a mean AUC 0 . 69 ± 0 . 0183, reflecting fair discriminatory ability but not accounting for censoring in survival data. SHAP analysis for the top-performing models highlighted the extent of lymph node involvement, Regional Node-Positive (number of affected lymph nodes), tumor grade (cell abnormality and growth rate), progesterone status, and age as key predictors of breast cancer survival outcomes.

**Conclusions** While ML models like XGBoost and RF can effectively identify important predictors and patterns in breast cancer outcomes, survival-specific methods such as the Cox model, RSF, and DeepSurv provide essential capabilities for handling time-to-event data and censoring, making them more suitable for accurate survival predictions. The primary objective of including ML models in this analysis was to leverage their interpretability in identifying key variables alongside survival-specific models, rather than to directly compare their performance against survival models. By examining both ML and survival models, this research highlights the complementary strengths of each approach. This study contributes to the integration of artificial intelligence in healthcare, emphasizing the value of data-driven survival modeling techniques in supporting healthcare professionals with accurate, personalized, and actionable insights for high-risk patients. Together, these approaches enhance the precision of survival predictions, paving the way for more informed clinical decision-making and improved patient care.

## Introduction

Breast cancer is one of the leading causes of death among women worldwide and remains a critical area of research due to its high incidence and mortality rates. In 2020, it became the most commonly diagnosed cancer globally, accounting for approximately 2.3 million new cases, or 11.7% of all cancer diagnoses [1]. In the United States alone, nearly 287, 850 women were diagnosed with breast cancer in 2022, resulting in 43, 250 deaths [2]. Survival rates show substantial variability, with five-year survival rates reaching around 80% in developed regions but often falling below 40% in developing countries, where healthcare resources are limited [3,4]. These disparities underscore the need for effective predictive models that can inform clinical decision-making, improve patient management, and ultimately enhance survival outcomes, particularly in resource-limited settings.

Survival analysis is a statistical approach widely used in such contexts to estimate the time until a specific event, such as death. It offers essential insights for patients and clinicians aiming to understand prognosis, especially in diseases with high mortality like breast cancer. This technique uniquely addresses censoring, where the exact time of an event remains unknown due to factors like loss to follow-up or a patient still alive at the study’s end [5]. Recently, machine learning (ML) models have emerged as valuable complements to traditional survival analysis, providing enhanced capabilities for diagnosis, prediction, and prognosis in medical applications. These methods support healthcare decision-making by enabling more accurate, data-driven risk assessments [6].

Various ML models have been applied to survival analysis in cancer research, with promising results. In one such study, [7] applied models like Multilayer Perceptron (MLP), Random Forest (RF), Decision Tree (DT), and Support Vector Machines (SVM) on a dataset of 4,902 patients, finding that MLP and RF achieved the highest accuracy, with rates of 88.2% and 83.3%, respectively. Similarly, [8] used Artificial Neural Networks (ANN) to predict 5-, 10-, and 15-year survival rates in 951 breast cancer patients, achieving high accuracy with AUC scores above 0.88 for each interval. In 2013, [9] analyzed 657,711 breast cancer patient data from surveillance, epidemiology, and end results (SEER) program and found that the C4.5 DT was the most effective algorithm for predicting ten-year survival. [10] compared the performance and stability of ten machine learning algorithms combined with eight feature selection methods for survival analysis of high-dimensional, heterogeneous clinical data. The results show that the ML algorithms performed well on the Sydney Memory and Ageing Study and the Alzheimer’s Disease Neuroimaging Initiative datasets.

Despite the popularity of these ML models, limitations arise in directly applying traditional ML methods to survival data due to their inability to account for censoring and time-to-event information, which are essential in survival analysis. This limitation often leads to biased or incomplete predictions, as traditional ML approaches struggle with handling time-dependent covariates and competing risks. In response, survival-specific ML methodologies, such as the Cox proportional hazard model (CPH), Random survival forest (RSF) and Cox proportional hazards deep neural network (DeepSurv), have been developed to address these limitations. RSF extends the traditional RF model [11–13] by incorporating censoring and time-dependent covariates, thus enhancing its applicability to survival data. DeepSurv applies a Cox proportional hazards model within a deep learning framework, enabling the modeling of nonlinear relationships between patient characteristics and survival outcomes, making it valuable for simulating complex clinical interactions [14]. One such study by [15] utilized these techniques and demonstrated that RSF outperforms both the CPH and Conditional Inference Forest (CIF) in modeling survival data. This finding was supported by prediction error curves and the C-index. Similarly, a study published by [14], which explored both simulated and real survival data, suggested that DeepSurv is an effective and powerful tool for predicting the probability of failure in patients and providing individualized treatment recommendations.

In light of this, the study examines traditional machine learning (ML) models alongside survival-specific approaches, such as CPH, RSF and DeepSurv, to understand their strengths in predicting breast cancer outcomes. Rather than focusing on direct performance comparisons, the emphasis is on the variables each model identifies as important, offering insights into their potential to enhance personalized treatment planning and prognostic tools for breast cancer patients.

The paper is organized as follows: we start with an overview of materials and methods, covering data sources, variables, and analytical techniques. This is followed by findings from the comparative analysis of predictive models. We conclude with a discussion of results, examining each approach’s strengths and limitations and suggesting directions for future research in breast cancer prognosis and machine learning applications.

## Materials and methods

### Data source and information

Patients diagnosed with infiltrating ductal and lobular carcinoma of the breast between 2006 and 2010 were accessed in April 2023 for research purposes. To enhance the reliability of the analysis, patients with missing data on tumor size, regional lymph nodes, regional positive lymph nodes, or those with survival durations of less than one month had been excluded, resulting in a final cohort of 4,024 individuals. The primary focus of the analysis was on survival time, measured in months, utilizing the survival months variable as the time-to-event metric and the status variable to indicate the occurrence of the event of interest (death) or censoring. The dataset included a range of clinical and demographic variables such as age, tumor stage (T, N, and A stages), tumor size, race, marital status, histological grade, hormone receptor status (estrogen and progesterone), and regional lymph node (positive and examined) involvement. Detailed descriptions of these variables are provided in S1 Table.

### Ethical considerations

This study used de-identified, publicly available data from the SEER program and was classified as exempt by the Institutional Review Board (IRB) at the University of Texas Rio Grande Valley (UTRGV) under reference number IRB-24-0400.

### Statistical analysis

This study utilized various statistical techniques to investigate the association between covariates and breast cancer survival. Relevant variables were selected based on a comprehensive review of prior literature to ensure their appropriateness for predictive modeling. Associations between categorical variables and survival status were examined using Pearson’s Chi-Squared Test or Fisher’s Exact Test, depending on the sample size and distribution within contingency tables. A significance level of *α* = 0 . 05 was used for all statistical tests. For the Bonferroni-adjusted pairwise comparisons, the significance threshold was calculated as $\frac{\alpha }{K}$, where *K* represents the number of comparisons made, ensuring a stringent control of the family-wise error rate. Collinearity among numerical variables was assessed using Spearman’s rank correlation to identify potential multicollinearity. For survival analysis, Kaplan-Meier (KM) estimates were used to generate survival curves, and global log-rank tests were performed to compare survival distributions between groups. Pairwise log-rank tests with Bonferroni-adjusted p-values were applied to identify specific group differences. To further explore the effect of specific covariates on overall survival, a multivariate Cox proportional hazards (CPH) model was utilized, enabling time-to-event analysis and assessment of statistical significance while controlling for multiple variables. Machine learning techniques were employed to identify key factors associated with breast cancer survival outcomes as they do not inherently account for censoring. Survival months was excluded from the ML analysis to avoid data leakage, as it directly reflects the predicted outcome. To address the class imbalance, the Synthetic Minority Oversampling Technique (SMOTE) [16] was applied, generating synthetic samples for the minority class to improve model robustness. SHapley Additive exPlanations values were used to interpret feature importance in RF, XGBoost, RSF, and DeepSurv models, providing insights into the contribution of variables to model predictions. Model performance was evaluated using multiple metrics, including mean accuracy, mean area under the curve (AUC), Concordance index (C-index), and Integrated Brier score (IBS), alongside their standard deviations. All statistical analyses and model implementations were conducted using the R language (version 4.3.1) [17] and Python (version 3.10.12) within the Google Colab environment.

### Summary description of data-driven survival models and machine learningmethods

**Random Survival Forests (RSF):** The RSF is implemented as an extension to the random forest that considers censored individuals. An advantage of this approach is that it is non-parametric and makes no assumption of the distribution functions. Here are the steps of the RSF as described by [11,12].

- Draw *B* bootstrap samples from the original data. Note that each bootstrap sample excludes on average 37% of the data, called out-of-bag (OOB) data. Here *B* represents the number of times the resampling procedure is performed and OOB data is the portion of the dataset excluded from a bootstrap sample during resampling. This OOB data is used for an unbiased assessment of the model’s performance on unseen data.
- Grow a tree for each bootstrapped dataset. At each node of the tree, randomly select *p* (number of predictor variables at a given split). A node is split on that predictor which maximizes survival differences across daughter nodes.
  For each node in a RSF, splitting rules determine the optimal division of data into child nodes. Two primary techniques: the log-rank and log-rank score split rules are used for this purpose, based on individuals’ survival times.
  The first split rule that is the log-rank, extends the traditional log-rank test to measure the difference in survival distributions of groups defined by each split. For a split at a value *c* of predictor *x*, the log-rank statistic *L* ( *x* , *c* )  is:
  $$\begin{array}{ccc} L(x,c)=\frac{\overset{N}{\underset{i=1}{\sum }}\left(d_{i,1}-Y_{i,1}\frac{d_{i}}{Y_{i}}\right)}{\sqrt{\overset{N}{\underset{i=1}{\sum }}\frac{Y_{i,1}}{Y_{i}}\left(1-\frac{Y_{i,1}}{Y_{i}}\right)\cdot \frac{Y_{i}-d_{i}}{Y_{i}-1}\cdot d_{i}}} & & \end{array}$$ (1)
  where $d_{i,1}$ is the number of events in group 1 at time *i*; $Y_{i,1}$ is the number at risk in group 1 at time *i*; $d_{i}$ is the total number of events at time *i*; and $Y_{i}$ is the total number at risk at time *i*.
  The split that maximizes  | *L* ( *x* , *c* ) |  across all potential splits is chosen, effectively distinguishing groups with significantly different survival patterns.
  The second split rule calculates the log-rank score *S* ( *x* , *c* )  based on survival time ranks. Assuming predictor *x* is ordered as $x_{1}\leq x_{2}\leq \cdots \leq x_{n}$, the rank for survival time $T_{l}$ is:
  $$\begin{array}{ccc} a_{l}=\delta_{l}-\overset{\Gamma_{l}}{\underset{k=1}{\sum }}\frac{\delta_{k}}{n-\Gamma_{k}+1} & & \end{array}$$ (2)
  where $\delta_{l}$ indicates an event at time $T_{l}$, and $\Gamma_{k}=\#\{t:T_{t}\leq T_{k}\}$ represents the count of times up to $T_{k}$.
  The log-rank score *S* ( *x* , *c* )  is then:
  $$\begin{array}{ccc} S(x,c)=\frac{\underset{x_{l}\leq c}{\sum }a_{l}-n_{1}\cdot ā}{\sqrt{n_{1}\left(1-\frac{n_{1}}{n}\right)s_{a}^{2}}} & & \end{array}$$ (3)
  where $n_{1}$ is the number of observations with $x_{l}\leq c$; *ā* is the mean of ranks $\left\{a_{l}\right\}$; and $s_{a}^{2}$ is the variance of ranks $\left\{a_{l}\right\}$. For further details on the properties of these split statistics, see [11,12].
- Grow the tree to full size under the constraint that a terminal node must have at least $d_{o}$ unique deaths (where $d_{o}$ is the minimum required number of unique death events per terminal node).
- Calculate a cumulative hazard function (CHF) for each tree. Average to obtain the ensemble CHF.
- Using OOB data, calculate the prediction error for the ensemble CHF.

The cumulative hazard function (CHF) for each terminal node *h* is estimated using the Nelson-Aalen estimator. This estimator accumulates the hazard contributions over time based on the number of deaths and individuals at risk at each event time within the terminal node. The formula for the CHF at time *t* is given by:


$$\begin{array}{ccc} {Ĥ}_{h}(t)=\underset{l:t_{l}\leq t}{\sum }\frac{d_{l,h}}{Y_{l,h}} & & \end{array}$$ (4)

where: ${Ĥ}_{h}(t)$ is the cumulative hazard for terminal node *h* at time *t*, $d_{l,h}$ is the number of deaths at event time $t_{l}$, $Y_{l,h}$ is the number of individuals at risk just before time $t_{l}$, The sum runs over all event times $t_{l}$ such that $t_{l}\leq t$. All individuals within a terminal node *h* share the same CHF, reflecting their similar survival experiences.

The final survival prediction is obtained by averaging the individual CHFs from all trees in the forest. The ensemble CHF at time *t* is:


$$\begin{array}{ccc} {Ĥ}_{\text{ensemble}}(t)=\frac{1}{B}\overset{B}{\underset{b=1}{\sum }}{Ĥ}_{b}(t) & & \end{array}$$ (5)

where: ${Ĥ}_{b}(t)$ is the CHF for the *b*-th tree in the forest, *B* here is the total number of trees in the forest.

**Cox Proportional Hazards (CPH) Model:** The CPH model is a semiparametric approach that models the hazard function, rather than survival times directly, to assess the effect of covariates on the risk of an event. To compare the relative hazards between two individuals *i* and *j* with covariates $Z_{i}$ and $Z_{j}$ at time *t*, the hazard ratio (HR) is given by:


$$\begin{array}{ccc} {\text{HR}}_{ij}=\frac{h_{i}(t,Z_{i})}{h_{j}(t,Z_{j})}=\exp\left(\overset{p}{\underset{k=1}{\sum }}\beta_{k}(Z_{ik}-Z_{jk})\right) & & \end{array}$$ (6)

This hazard ratio provides insights into the relative risk associated with different covariates [18,19]. A detailed explanation of the model, including the mathematical formulation and interpretation, is provided in S1 Appendix.

**Cox Proportional Hazard Deep Neural Network (DeepSurv):** DeepSurv proposed by [14] as an extension of the Farragi and Simon architecture [20], is a deep feed-forward neural network for survival analysis based on a multi-layer perceptron (MLP). DeepSurv learns relationships between input features and the hazard rate through hidden layers and estimates the log-risk function associated with the CPH model in the output layer. The model’s loss function is derived from the negative log partial likelihood of the Cox model, allowing it to estimate hazard ratios while learning complex patterns in the data. To prevent overfitting, $L_{2}$ regularization is applied to the network weights. Details on the partial likelihood and loss function are provided in S1 Appendix.

**Benchmark ML Models:** Several machine learning (ML) models were selected as benchmarks in this study, focusing on variable importance related to breast cancer survival. These ML models are widely used in biomedical research due to their strong predictive performance and ability to identify influential features. Although these models do not account for time-to-event data or censoring, a limitation often highlighted in survival analysis literature, they are valuable for preliminary feature selection and can provide insights into variable importance when used alongside survival-specific models. The selected ML models include XGBoost, a gradient boosting algorithm that aggregates multiple weak learners, typically decision trees, to enhance predictive accuracy [21]; Light Gradient Boosting method (LGBM), which similarly uses gradient boosting but is optimized for large datasets via a leaf-wise growth strategy, improving scalability [22]; SVM with an RBF kernel, designed to maximize class separation through an optimal hyperplane, thus improving classification accuracy [23,24]; and RF, an ensemble model that combines multiple decision trees to reduce variance and improve prediction stability [13]. This complementary approach aims to leverage the ML models’ strength in identifying key predictors while addressing their limitations in handling censored survival data through the use of survival-specific models, thus supporting a more robust analysis of breast cancer prognosis.

### Model tuning and validation

The dataset, consisting of 4,024 records, was divided into a training set (80%, 3,219 records) and a hold-out test set (20%, 805 records) for final evaluation. Within the training data, model-specific strategies were employed for validation and hyperparameter optimization. For the DeepSurv model, 20% (644 records) of the training set was reserved as a validation set. This subset was used to guide model tuning and monitor performance for early stopping. The DeepSurv model was fine-tuned using hyperparameters such as the number of layers, dropout rate, and learning rate, optimized with Optuna [25]. To further ensure robustness, 10-fold cross-validation was also conducted on the training set. In contrast, the Cox Proportional Hazards (CPH) and Random Survival Forest (RSF) models utilized 5-fold cross-validation across the training data for both performance assessment and hyperparameter tuning. For RSF, hyperparameters such as the number of trees, the number of variables considered at each split, and the minimum node size were optimized. Additionally, two splitting criteria—logrank and logrank score were explored to identify the optimal configuration. Other machine learning models followed a similar tuning process, with hyperparameters optimized using either grid search or randomized search, each integrated with 5-fold cross-validation. All models—DeepSurv, CPH, RSF, and the ML techniques were ultimately evaluated on the independent test set to ensure reliability and generalizability.

### Model evaluation metrics and explainability

1. **Harrel’s Concordance index (C-index)**: The C-index measures how well a model correctly ranks the survival times of individuals, with values ranging from 0.5 to 1. A value of 0.5 indicates random chance, whereas values above 0.7 suggest good model performance, with values exceeding 0.8 indicative of strong predictive capability. A value of 1 represents perfect prediction. For more details, refer to [12].
2. **Integrated Brier Score (IBS)**: The Brier score evaluates the accuracy of survival function predictions at a specific time *t*, while the Integrated Brier Score (IBS) assesses overall model performance across all times, with values ranging from 0 to 1 (0 being ideal). For more details, see [26,27].
3. **Shapley Additive Explanations (SHAP)**: SHAP values were used to measure the contribution of individual features to the model’s predictions [28,29]. A detailed explanation of SHAP, including its mathematical formulation, is provided in S1 Appendix.

## Results

Table 1 presents baseline characteristics for 4,024 breast cancer patients, stratified by survival status (censored vs. deceased). Age distribution was similar across groups, with the largest proportion in the 30-50 year range (37%). Tumor size differed significantly (*p* < 0 . 001), with smaller tumors ( < 25 mm) more common in censored patients (52%) and larger tumors ( > 45 mm) more frequent in deceased patients (27%). T Stage, (*p* < 0 . 001) and grade (*p* < 0 . 001) showed significant differences, with early-stage disease (especially T2) and Grade II being prevalent among censored patients, while advanced stages and grades were more common in deceased patients. Hormone receptor status also varied significantly, with estrogen and progesterone positivity more frequent in censored patients (*p* < 0 . 001). While White patients made up 85% of the population, race distribution still differed significantly (*p* < 0 . 001), with Black patients more represented among the deceased (12% vs. 6.4%). Marital status differed (*p* < 0 . 001), with married patients predominantly in the censored group (67%), while divorced and widowed patients were more frequent among the deceased. Significant associations were observed between survival status (deceased and censored) and all variable categories, highlighting their relationship with patient outcomes.

**Table 1:** Summary of baseline characteristics in breast cancer patients, stratified by survival status (censored vs deceased).

Variable	Overall (N = 4,024)	Censored (N = 3,408)	Deceased (N = 616)	p-value^*2*^
**Age Category, n (%)**				< 0.001
30-50	1,498 (37%)	1,288 (38%)	210 (34%)	
51-60	1,386 (34%)	1,209 (35%)	177 (29%)	
61-70	1,140 (28%)	911 (27%)	229 (37%)	
**Tumor Size Category, n (%)**				< 0.001
< 25 mm	1,990 (49%)	1,768 (52%)	222 (36%)	
25-45 mm	1,321 (33%)	1,094 (32%)	227 (37%)	
> 45 mm	713 (18%)	546 (16%)	167 (27%)	
**Race, n (%)**				< 0.001
Black	291 (7.2%)	218 (6.4%)	73 (12%)	
Other	320 (8.0%)	287 (8.4%)	33 (5.4%)	
White	3,413 (85%)	2,903 (85%)	510 (83%)	
**Marital Status, n (%)**				< 0.001
Divorced	486 (12%)	396 (12%)	90 (15%)	
Married	2,643 (66%)	2,285 (67%)	358 (58%)	
Separated	45 (1.1%)	30 (0.9%)	15 (2.4%)	
Single	615 (15%)	511 (15%)	104 (17%)	
Widowed	235 (5.8%)	186 (5.5%)	49 (8.0%)	
**T Stage, n (%)**				< 0.001
T1	1,603 (40%)	1,446 (42%)	157 (25%)	
T2	1,786 (44%)	1,483 (44%)	303 (49%)	
T3	533 (13%)	417 (12%)	116 (19%)	
T4	102 (2.5%)	62 (1.8%)	40 (6.5%)	
**Grade, n (%)**				< 0.001
Grade I	543 (13%)	504 (15%)	39 (6.3%)	
Grade II	2,351 (58%)	2,046 (60%)	305 (50%)	
Grade III	1,111 (28%)	848 (25%)	263 (43%)	
Grade IV	19 (0.5%)	10 (0.3%)	9 (1.5%)	
**Estrogen Status, n (%)**				< 0.001
Negative	269 (6.7%)	161 (4.7%)	108 (18%)	
Positive	3,755 (93%)	3,247 (95%)	508 (82%)	
**Progesterone Status, n (%)**				< 0.001
Negative	698 (17%)	494 (14%)	204 (33%)	
Positive	3,326 (83%)	2,914 (86%)	412 (67%)	

^1^ categorical variables as n (%).

^2^ P-values are calculated using Pearson’s Chi-Squared Test or Fisher’s Exact Test where appropriate.

### Kaplan-meier (KM) analysis

**Fig 1:**
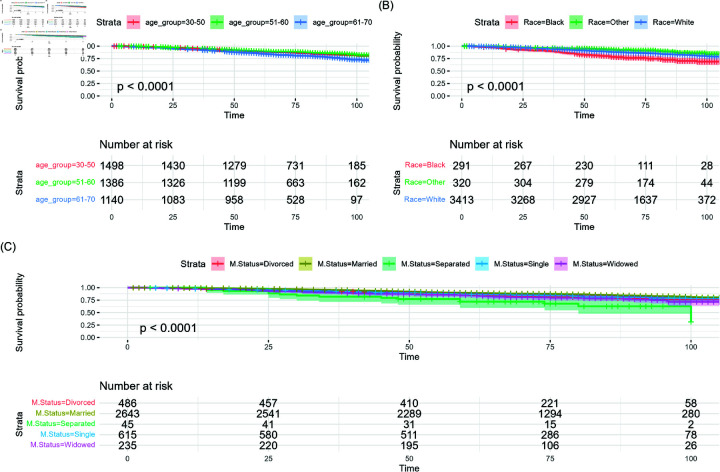
Kaplan-Meier survival curves of demographic factors on the overall dataset  ( *N* = 4 , 024 )  of breast cancer patients with pairwise comparisons. (A) KM curve for age groups and the associated risk table. (B) KM curve for race and associated risk table. (C) KM curve for marital status and associated risk table.

In the study, Kaplan-Meier (KM) survival curves were employed to evaluate the influence of demographic and clinical factors on breast cancer survival. Fig 1 shows that patients aged 61-70 had significantly lower survival probabilities over time compared to the 30-50 and 51-60 age groups, and also highlights racial disparities, with Black patients exhibiting lower survival rates than their White counterparts. Additionally, separated individuals showed reduced survival chances in comparison to married individuals.

Pairwise log-rank tests with Bonferroni correction confirmed significant survival differences between age groups 30-50 and 61-70, and 50-60 and 61-70 (*p* = 0 . 0002, *p* < 0 . 0001). Significant racial differences in survival were observed, with both Black vs. White and Black vs. Other comparisons showing significance after Bonferroni correction (*p* < 0 . 001). No significant difference was found between White and Other racial groups (*p* = 0 . 08). Marital status was significantly associated with survival, with only the comparison between separated and married patients remaining significant after Bonferroni correction (*p* = 0 . 0001). Other comparisons (married vs. divorced or widowed) did not reach the adjusted significance threshold. The results highlight the impact of age, race, and marital status on breast cancer survival, with older patients, Black individuals, and separated patients experiencing lower survival. Prior studies have also reported that younger Black women and certain minority groups face higher breast cancer risks and mortality at younger ages [30,31].

**Fig 2:**
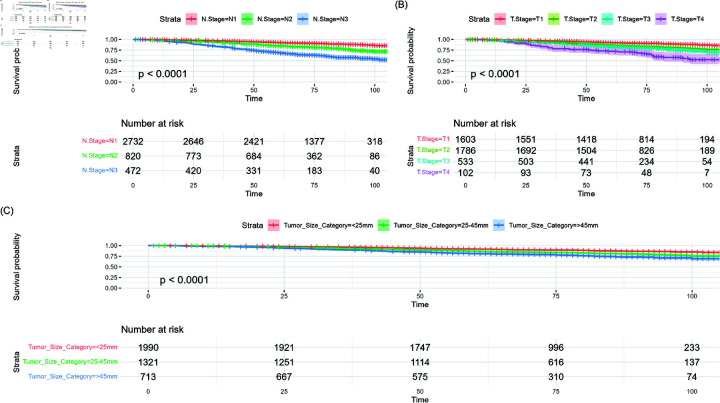
Kaplan-Meier survival curves of clinical factors on the overall dataset  ( *N* = 4 , 024 )  of breast cancer patients with pairwise comparisons. (A) KM for N stage and associated risk table. (B) KM for T stage and associated risk table. (C) KM curve for tumor size and associated risk table.

From Fig 2, Patients with N3 and T4 stages of breast cancer had lower survival probabilities compared to those with N1 and T1 stages. Additionally, tumors smaller than 25 mm were associated with higher survival probabilities than tumors larger than 45 mm. In terms of the N Stage, all three stages showed statistically significant differences in survival curves (*p* < 0 . 0001). However, for the T Stage, no significant difference was observed between the survival curves of T2 and T3 (*p* = 0 . 0726). There were significant differences in survival between the tumor size groups of less than 25 mm and 25-45 mm (*p* < 0 . 0001), as well as between the groups of less than 25 mm and greater than 45 mm (*p* < 0 . 0001), as determined by Bonferroni-adjusted p-values. Additionally, there was a significant difference in survival between the 25-45 mm and greater than 45 mm groups (*p* = 0 . 002).

### RSF analysis

**Fig 3:**
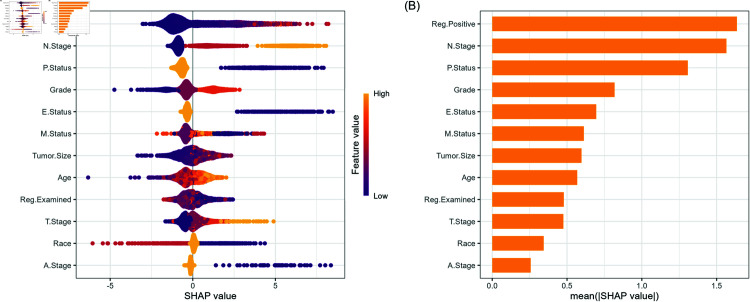
Feature importance and beeswarm plot determined by mean absolute SHAP values for the random survival forest (RSF) model. (A) SHAP value distribution for features, highlighting the relationship between feature values and their impact on model predictions. (B) Bar plot of mean absolute SHAP values by decreasing order of importance.

Both log-rank and log-rank score splitting criteria were considered, with the log-rank score criterion selected for its ability to minimize the mean error rate, resulting in more precise and stable survival predictions. An error rate plot against the number of trees was generated to determine the optimal number of trees required for robust performance (See S1 Fig). The SHAP plot in Fig 3 shows the impact of key features on breast cancer survival predictions. Regional node Positive, representing the number of positive lymph nodes, is a top predictor, with higher values linked to worse survival outcomes, aligning with clinical expectations. Similarly, the N Stage, indicating the extent of lymph node involvement, also negatively affects survival as it increases. Other influential features, like Progesterone status, Grade, Estrogen status, Marital status and Tumor size, further contribute to the model’s predictions. This plot highlights how each factor shapes the survival forecast, emphasizing the importance of lymph node involvement and tumor severity in prognostic modeling.

The optimized RSF model generated survival curves based on predictions for different age groups and tumor sizes, both of which are crucial prognostic factors in breast cancer. As shown in S2 Fig, age had a modest impact on survival probability, with the oldest cohort (age 61-70 years) demonstrating a slightly steeper decline in survival after 60 months, highlighting the increased risk associated with older age. The survival curves for the age groups 30-50 and 51-60 were relatively similar, suggesting that other factors may play a more significant role in influencing survival outcomes within these age groups. Additionally, patients with smaller tumors (Low,  < 25mm) had higher survival probabilities compared to those with Medium (25 − 45mm) or High ( > 45mm) tumors. This suggests that tumor size remains a significant determinant of survival, with smaller tumors being associated with better prognosis. These survival curves provide valuable insights into the relationship between tumor size, age, and patient survival and should be integrated with clinical expertise when making treatment decisions, ensuring a more personalized approach to patient care.

### CPH analysis

According to Table 2, the analysis shows that every additional year of age is associated with a 2% increase in mortality risk (HR = 1.02, *p* < 0 . 0001). Additionally, having positive regional nodes is linked with a 7% increase in mortality risk (HR = 1.07, *p* < 0 . 0001), while the number of regional nodes examined correlates with a 3% decrease in mortality risk (HR = 0.97, *p* < 0 . 0001). Regarding race, compared to black patients, white patients have a 30% lower hazard of mortality (HR = 0.70, *p* = 0 . 014), and patients of other races have a 49% lower hazard of mortality (HR = 0.51, *p* = 0 . 004).

**Table 2:** Results of the multivariate analysis of the CPH model after stratification for key variables.

Analysis Variables	Categories	HR	95% CI	P-value
Age		1.02	1.01 - 1.03	< 0.0001*
Regional node Positive		1.07	1.04 - 1.09	< 0.0001*
Regional node Examined		0.97	0.96 - 0.98	< 0.0001*
Race	Black	-		
	White	0.70	0.52-0.92	0.014*
	Other	0.51	0.31 - 0.80	0.004*
Grade	I	-		
	II	1.66	1.13 - 2.43	0.009*
	III	2.48	1.67 - 3.66	< 0.0001*
	IV	5.61	2.61 - 12.08	< 0.0001*
Tumor size		1.00	0.99 - 1.00	0.990
T Stage	T1	-		
	T2	1.47	1.14 - 1.89	0.003*
	T3	1.58	0.98 - 2.55	0.059
	T4	2.17	1.32 - 3.56	0.002*
N Stage	N1	-		
	N2	1.60	1.26 - 2.03	0.0001*
	N3	1.66	1.11 - 2.44	0.011*
Marital Status	Divorced	-		
	Married	0.74	0.57 - 0.95	0.022*
	Single	0.89	0.65- 1.21	0.464
	Widowed	0.88	0.58 - 1.30	0.512
	Separated	1.39	0.68 - 2.80	0.357

HR: Hazard Ratio, 95% CI shows the lower and upper bound of the 95% Confidence Interval. Estrogen Status, Progesterone Status, and A Stage were stratified in the model.

Using Grade I as a reference, patients with Grade IV (the most abnormal cells) faced a 5.61-fold increase in mortality risk (*p* < 0 . 0001). Grade III showed a 2.48-fold increase (*p* < 0 . 0001), while Grade II presented a 66% higher risk (HR = 1.66, *p* = 0 . 009). In terms of T Stage, compared to T1, T2 was associated with a 47% increased mortality risk (HR = 1.47, *p* = 0 . 003), and T4 showed a 2.17-fold increase in mortality risk (*p* = 0 . 002). For N Stage, N2 was linked to a 60% higher mortality risk (HR = 1.60, *p* = 0 . 0001), and N3 to a 66% increased risk (HR = 1.66, *p* = 0 . 011). Regarding marital status, separated patients exhibited a 1.39-fold higher mortality risk, though this was not statistically significant (*p* = 0 . 357), while married patients showed a 26% lower hazard of mortality (HR = 0.74, *p* = 0 . 022).

The performance of the Cox model was evaluated using the C-index and IBS. The Cox model achieved a C-index of 0.71 ( ±  0.015), indicating strong discriminative ability, and an IBS of 0.082 ( ± 0 . 006), reflecting accurate and reliable survival probability predictions over time. The proportional hazards assumption was tested using the Schoenfeld residual test [32] to assess covariates for proportionality. Covariates that violated this assumption (*p* < 0 . 05) were stratified to account for non-proportional hazards. Estrogen Status, Progesterone Status, and A Stage were included as stratification factors in the Cox proportional hazards model, allowing different baseline hazards for these variables. The multivariate Cox analysis identified several factors influencing breast cancer prognosis. Poorer survival outcomes were associated with older age, higher tumor grade (II, III, IV), advanced T stage (T2, T3, T4), and increased lymph node involvement (N2, N3), reflecting more aggressive disease. A higher count of positive lymph nodes also indicated a worse prognosis.

In contrast, examining lymph nodes, being of White or Other race, and being married were linked to better survival. These factors likely reflect improved staging, healthcare access, and social support. The multivariate Cox proportional hazards analysis and its findings are visually summarized in the forest plot (see S3 Fig).

### DeepSurv analysis

**Fig 4:**
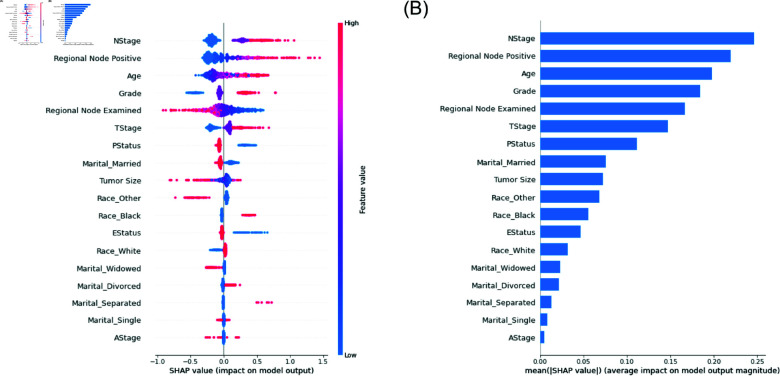
Feature importance and beeswarm plot determined by mean absolute SHAP values for the DeepSurv model. (A) A comprehensive view of the influence each variable has on model predictions, with N Stage Status ranked on top (B) Bar plot of mean absolute SHAP values by decreasing order of importance

The optimal configuration of 4 hidden layers with 64 neurons each and a 20% dropout rate was identified. The SHAP summary plot in Fig 4 identified N Stage, Regional node Positive, Age, Grade, Regional node examined, and T Stage as significant factors in breast cancer prognosis. Notably, higher N Stage, Age, Grade and Regional node positive were associated with increased risk, aligning with clinical expectations and reinforcing the model’s relevance in predicting breast cancer survival outcomes. The training and validation loss plot is shown in S4 Fig.

### Analysis of benchmark machine learning models

**Fig 5:**
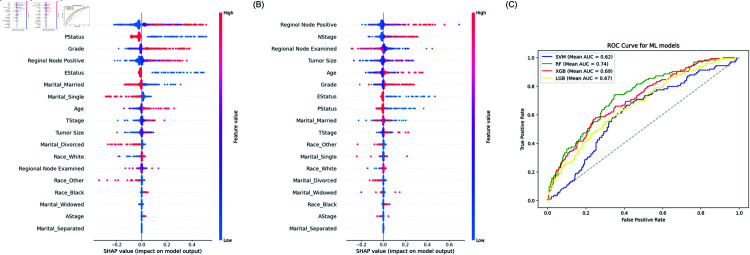
Bee-swarm visualization, sorted by the mean absolute SHAP values, showcasing the variables’ influence on the models’ predictions. (A) SHAP feature Importance of the XGBoost model. (B) SHAP feature Importance of the RF model. (C) Comparison of ROC-AUC Scores of the ML models.

As the XGBoost and RF models performed better than the other ML models, they were further examined using the SHAP framework. The most important variables are displayed for both the XGBoost algorithm and the RF model. The covariates are displayed according to their level of relevance on the y-axis, while the SHAP values on the x-axis illustrate the impact of the variable on the output of the XGBoost and RF model. Fig 5 shows that N Stage, Progesterone Status, Regional node-positive, Grade, Estrogen Status, Tumor size, Regional node examined, and Age appear as some top contributors in the two models.

Table 3 compares the performance of the various models using metrics such as accuracy, AUC, C-index, and IBS. Machine learning models, such as XGBoost (mean accuracy = 0 . 84 ± 0 . 0035, AUC = 0 . 69 ± 0 . 0183) and RF (mean accuracy = 0 . 82 ± 0 . 0018, AUC = 0 . 74 ± 0 . 0021), demonstrated strong classification performance, attributable to their ability to capture complex, non-linear relationships and feature interactions within the dataset. XGBoost’s gradient-boosting framework iteratively minimizes classification errors, while RF leverages an ensemble of decision trees to mitigate overfitting. These capabilities are particularly advantageous in datasets with heterogeneous characteristics, allowing these models to highlight critical prognostic factors and stratify patients effectively. However, ML models do not explicitly account for survival time or censoring, which limits their application to survival analysis. Their strong accuracy and AUC metrics reflect their proficiency in classification tasks, but these measures may not fully translate to survival prediction.

**Table 3:** Comparison of models employed with their respective performance measures.

		mean Accuracy ( ± SD)	mean AUC/ C-index ( ± SD)	IBS ( ± SD)
ML	XGBoost	0.84 ± 0 . 0035	0 . 69 ± 0 . 0183	
	RF	0.82 ± 0 . 0018	0 . 74 ± 0 . 0021	
	SVM	0.77 ± 0.0000	0.62 ± 0.0001	
	LGBM	0.83 ± 0 . 0042	0.67 ± 0.0096	
Survival models	CPH		0 . 71 ± 0 . 015	0.08 ± 0 . 006
	RSF		0 . 72 ± 0 . 0117	0 . 08 ± 0 . 0024
	DeepSurv		0 . 71 ± 0 . 0095	0 . 09 ± 0 . 0008

Survival-specific models such as Cox, RSF, and DeepSurv are designed to predict time-to-event outcomes while handling censoring, making them particularly suitable for survival analysis. Among these, RSF achieved the highest C-index (0 . 72 ± 0 . 0117), slightly outperforming both Cox (0 . 71 ± 0 . 015) and DeepSurv (0 . 71 ± 0 . 0095). RSF’s superior ranking ability can be attributed to its capacity to model non-linear relationships and interactions, which is especially beneficial in datasets with diverse clinical characteristics. In terms of survival probability estimation, all three models demonstrated comparable accuracy over time. RSF and Cox achieved the lowest IBS (0 . 08), with DeepSurv following closely (0 . 09). These minor differences in IBS suggest limited practical impact. The observed variations in model performance reflect differences in their underlying methodologies and the nature of the breast cancer dataset. ML models excel at identifying complex patterns and interactions, survival-specific models address the time component of survival analysis, providing more reliable survival estimates and event rankings. Ultimately, the choice of model depends on the analytical goals. ML models are powerful for identifying key predictors and stratifying risk groups, whereas survival-specific models are essential for accurate time-to-event analyses. These findings highlight the complementary strengths of the two approaches, emphasizing the importance of aligning model selection with the dataset characteristics and research objectives.

### Discussion and conclusion

This study aimed to enhance understanding of breast cancer prognosis by evaluating multiple machine learning (ML) and survival-specific models, each offering unique insights into survival prediction. ML models, such as XGBoost and Random Forest (RF), excel at identifying non-linear relationships and interactions within clinical and demographic data, highlighting critical predictors linked to various diseases. While these models do not directly account for survival time or censoring, their strong performance in metrics like accuracy and AUC demonstrates their utility in identifying prognostic factors.

In contrast, survival models—CPH, RSF, and DeepSurv—are designed to handle censored data and predict time-to-event outcomes. The RSF model’s ability to capture non-linear effects and interactions enhances its suitability for patients with diverse or atypical characteristics, supporting personalized treatment planning. DeepSurv, a deep learning-based model, excels in uncovering complex relationships within data, making it particularly valuable for datasets containing genetic or molecular information. SHAP analysis revealed consistent predictors of breast cancer outcomes. Across most models, factors such as Lymph node stage, Regional node positive, Grade, Progesterone status, and Age emerged as critical predictors, aligning with their established roles as markers of cancer progression and prognosis. Estrogen receptor status was also a relevant predictor in most models, though with slightly reduced influence in DeepSurv. While core clinical variables remained consistent, each model highlighted additional aspects. RSF placed greater emphasis on hormonal markers, particularly progesterone and estrogen receptor status, underscoring their relevance in hormone-sensitive disease. DeepSurv gave more weight to marital status, suggesting a potential influence of social support on outcomes. The machine learning models further emphasized socio-demographic variables, including race and specific marital categories, pointing to broader determinants such as healthcare access and social context.

These findings highlight the complementary strengths of different modeling techniques. The shared importance of well-established clinical features supports their continued use in risk stratification and treatment planning. Meanwhile, the additional insights from social and demographic variables reflect the potential value of integrating holistic, patient-centered factors into prognostic models. Incorporating such approaches into clinical practice may improve personalized care and support efforts to reduce disparities in breast cancer outcomes.

Despite these promising results, this study has limitations. The exclusion of genetic, molecular, and lifestyle data narrows the scope of analyzed predictors. Future research should incorporate these data types and explore multi-modal settings that integrate imaging, genomics, and clinical data to enhance predictive power and clinical applicability. Prospective validation in diverse patient populations is also crucial to ensure generalizability. This study underscores the value of combining diverse survival modeling techniques to achieve a comprehensive understanding of breast cancer prognosis. Consistent identification of key predictors validates their clinical relevance, while the unique insights from each model offer avenues for further exploration. With richer data integration, these approaches hold significant promise for translating research findings into meaningful clinical applications, ultimately improving risk assessment, personalized treatment, and patient outcomes.

## Supporting information

S1 Appendix Descriptions of CPH, DeepSurv and SHAP Framework (PDF)

S1 Fig Error rate plot for RSF model with increasing number of trees. (PDF)

S2 Fig (A) Predicted survival probabilities for different age size groups with the optimal RSF model. (B) Predicted survival probabilities for different tumor size groups with the optimal RSF model.
(PDF)

S3 Fig Multivariate CPH analysis – HR’s with 95% CI. Variables of Higher HR’s are sorted in descending order.*p < 0 . 05, **p < 0 . 01 ,***p < 0 . 001.
(PDF)

S4 Fig Training and validation loss plot for the DeepSurv model. (PDF)

S1 Table Breast Cancer Patient Information (PDF)
