# Supplementary material for: Data-driven survival modeling for breast cancer prognostics: A comparative study with machine learning and traditional survival modeling methods
Source: PLoS One. 2025 Apr 22;20(4):e0318167. doi: 10.1371/journal.pone.0318167 (PMC12014147; doi:10.1371/journal.pone.0318167)
Supplement: S1 Appendix — (PDF) [file pone.0318167.s001.pdf]

Data-Driven Survival Modeling for Breast Cancer Prognostics: A Comparative Study with Machine Learning and Traditional Survival Modeling Methods

Theophilus Gyedu Baidoo [1], Hansapani Rodrigo[1]

1 School of Statistical and Mathematical Sciences, The University of Texas Rio Grande Valley, Edinburg, Texas, United States of America

*Corresponding Author: hansapani.rodrigo@utrgv.edu

A Appendix

A.1 Cox Proportional Hazards (CPH) Model

The Cox Proportional Hazards (CPH) model is a semiparametric approach that models the hazard function, rather than survival times directly, to assess the effect of covariates on the risk of an event. The model is defined as follows:

$$h_i(t, Z_i) = h_0(t) \exp\left(\sum_{k=1}^{p} \beta_k Z_{ik}\right) \tag{1}$$

where:

- $h_i(t, Z_i)$: Hazard function for individual i at time t conditioned on covariates Z_i,
- $h_0(t)$: Baseline hazard (hazard when all covariates are zero),
- β_k: Coefficient for covariate Z_{ik},
- p: Total number of covariates.

Interpretation of the hazard ratio:

- HR > 1: Increased risk for individual i relative to j,
- HR < 1: Reduced risk,
- HR $= 1$: No difference in risk, assuming other factors remain constant.

The semiparametric nature of the model allows flexible estimation of the baseline hazard while quantifying covariate effects, making it a valuable tool for analyzing breast cancer survival risk factors.

A.2 DeepSurv Partial Likelihood and Loss Function

In DeepSurv, the partial likelihood of the Cox model, given a set of censored event times $c_1, \ldots, c_k$, is defined as:

$$L_{cox} = \prod_{k=1}^{n} \left(\frac{e^{\beta_k Z_k(c_k)}}{\sum_{j \in R_k} e^{\beta_k Z_k(c_k)}} \right)^{D_k} \tag{2}$$

Here, R_k represents the set of patients at risk at each event time, and D_k indicates whether an event was observed for patient k.

The loss function in DeepSurv is derived by taking the negative log of the partial likelihood:

$$loss(\theta) = -\frac{1}{n(D=1)} \sum_{k:D_k=1} \log \left(\sum_{j \in R_k} \exp(\beta_k Z_k(c_k)) - \beta_k Z_k(c_k) \right) + \lambda \cdot ||\theta||_2^2 \tag{3}$$

Here, λ is the regularization parameter, balancing fit and prevention of overfitting by penalizing large parameter values.

A.3 Shapley Additive Explanations (SHAP)

SHAP values provide a consistent framework to measure the impact of each feature on a model's predictions by attributing portions of the model's output to individual features. Derived from Shapley values in cooperative game theory, SHAP calculates the contribution of feature j as:

$$\phi_j^S = \frac{1}{K} \sum_{i=1}^{K} \left[f(x_i) - \frac{1}{|S_{i,j}|} \sum_{s \in S_{i,j}} f(z_{i,j}^S) \right] \tag{4}$$

where:

- ϕ_j^S: SHAP value representing the contribution of feature j within data subset S,
- $f(x_i)$: Model prediction for sample x_i,
- $z_{i,j}^S$: A new sample where feature j in x_i is replaced with the value from another sample,
- $S_{i,j}$: Subset of samples used for feature replacement.

where ϕ_j^S is the SHAP value representing the contribution of feature j within data subset S. For each sample x_i, a new version $z_{i,j}^S$ is generated by replacing the value of feature j with one from another sample, allowing the model to compute the difference in output. Averaging these differences across samples yields the SHAP value ϕ_j^S, capturing the importance of feature j.
