# Supplementary material for: Data-driven survival modeling for breast cancer prognostics: A comparative study with machine learning and traditional survival modeling methods
Source: PLoS One. 2025 Apr 22;20(4):e0318167. doi: 10.1371/journal.pone.0318167 (PMC12014147; doi:10.1371/journal.pone.0318167)
Supplement: S2 Fig — (B) Predicted survival probabilities for different tumor size groups with the optimal RSF model. (PDF) [file pone.0318167.s003.pdf]

Data-Driven Survival Modeling for Breast Cancer Prognostics: A Comparative Study with Machine Learning and Traditional Survival Modeling Methods

Theophilus Gyedu Baidoo [1], Hansapani Rodrigo[1]

1 School of Statistical and Mathematical Sciences, The University of Texas Rio Grande Valley, Edinburg, Texas, United States of America

*Corresponding Author: hansapani.rodrigo@utrgv.edu

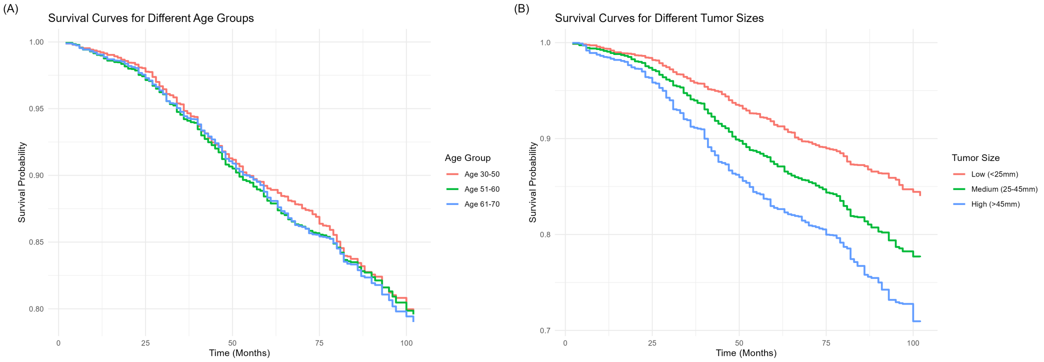

S2 Fig . **(A)** Predicted Survival Probabilities for different age size groups with the optimal RSF model. **(B)** Predicted Survival Probabilities for different tumor size groups with the optimal RSF model.
