# Supplementary material for: Data-driven survival modeling for breast cancer prognostics: A comparative study with machine learning and traditional survival modeling methods
Source: PLoS One. 2025 Apr 22;20(4):e0318167. doi: 10.1371/journal.pone.0318167 (PMC12014147; doi:10.1371/journal.pone.0318167)
Supplement: S1 Table — (PDF) [file pone.0318167.s006.pdf]

Data-Driven Survival Modeling for Breast Cancer Prognostics: A Comparative Study with Machine Learning and Traditional Survival Modeling Methods

Theophilus Gyedu Baidoo [1], Hansapani Rodrigo[1]

1 School of Statistical and Mathematical Sciences, The University of Texas Rio Grande Valley, Edinburg, Texas, United States of America

*Corresponding Author: hansapani.rodrigo@utrgv.edu

Table 1. Clinical variables of breast cancer and its description

Variables	Description
Age of Patients	Age at the time of diagnosis is categorized in three groups.
Race	numbered 1 to 3, 1 for white, 2 for Black, and 3 for other (American Indian/AK Native, Asian/Pacific Islander)
Marital Status	numbered 1 to 5, 1 for divorced, 2 for married, 3 for Separated, 4 for Single, and 5 for Widowed.
T Stage	labeled 1 to 4, 1 for T1, 2 for T2, 3 for T3, and 4 for T4. The higher the number after the T, the larger the tumor or the more it has grown into nearby tissues.
N Stage	labeled 1 to 3, 1 for N1, 2 for N2, 3 for N3. The higher the number after the N, the more lymph node contains cancer.
A stage	a simplified version of the stage to check the cancer spread. It is a historical staging system that takes on Regional and Distant in this dataset. Regional means that cancer has spread from the breast to nearby nodes/structures. Distant means that cancer has spread from the breast to distant parts of the body, such as the bones, liver, or lungs.
Grade	I- Tumor cells and tissue looks most like healthy cells and tissue. These are called well-differentiated tumors and are considered low-grade. II-The cells and tissue are somewhat abnormal and are called moderately differentiated. III- Cancer cells and tissue look very abnormal. These cancers are considered poorly differentiated since they no longer have an architectural structure or pattern. IV- These undifferentiated cancers have the most abnormal-looking cells. These are the highest grade and typically grow and spread faster than lower-grade tumors.
Tumor size	indicating the size of the tumor and categorized into three groups. Measured in mm.
Survival time	time to death measured in months.
Regional nodes examined	Records the total number of regional lymph nodes that were removed and examined by the pathologist.
Regional node positive	Records the exact number of regional nodes examined by the pathologist and found to contain metastases
Estrogen status	labeled positive and Negative if cancer has receptors for estrogen or not.
Progesterone status	labeled positive or negative if cancer has progesterone receptors or not.
Status	the event indicator, 1 for patients who deceased before the end of the study, and 0 for patients who were censored.
